# Lifelong Learning of Spatiotemporal Representations With Dual-Memory Recurrent Self-Organization

**DOI:** 10.3389/fnbot.2018.00078

**Published:** 2018-11-28

**Authors:** German I. Parisi, Jun Tani, Cornelius Weber, Stefan Wermter

**Affiliations:** ^1^Knowledge Technology, Department of Informatics, Universität Hamburg, Hamburg, Germany; ^2^Cognitive Neurorobotics Research Unit, Okinawa Institute of Science and Technology, Okinawa, Japan

**Keywords:** lifelong learning, complementary learning systems, self-organizing networks, continuous object recognition, catastrophic forgetting

## Abstract

Artificial autonomous agents and robots interacting in complex environments are required to continually acquire and fine-tune knowledge over sustained periods of time. The ability to learn from continuous streams of information is referred to as *lifelong learning* and represents a long-standing challenge for neural network models due to *catastrophic forgetting* in which novel sensory experience interferes with existing representations and leads to abrupt decreases in the performance on previously acquired knowledge. Computational models of lifelong learning typically alleviate catastrophic forgetting in experimental scenarios with given datasets of static images and limited complexity, thereby differing significantly from the conditions artificial agents are exposed to. In more natural settings, sequential information may become progressively available over time and access to previous experience may be restricted. Therefore, specialized neural network mechanisms are required that adapt to novel sequential experience while preventing disruptive interference with existing representations. In this paper, we propose a dual-memory self-organizing architecture for lifelong learning scenarios. The architecture comprises two growing recurrent networks with the complementary tasks of learning object instances (episodic memory) and categories (semantic memory). Both growing networks can expand in response to novel sensory experience: the episodic memory learns fine-grained spatiotemporal representations of object instances in an unsupervised fashion while the semantic memory uses task-relevant signals to regulate structural plasticity levels and develop more compact representations from episodic experience. For the consolidation of knowledge in the absence of external sensory input, the episodic memory periodically replays trajectories of neural reactivations. We evaluate the proposed model on the CORe50 benchmark dataset for continuous object recognition, showing that we significantly outperform current methods of lifelong learning in three different incremental learning scenarios.

## 1. Introduction

Artificial autonomous agents and robots interacting in dynamic environments are required to continually acquire and fine-tune their knowledge over time (Thrun and Mitchell, 1995; Parisi et al., 2018a). The ability to progressively learn over a sustained time span by accommodating novel knowledge while retaining previously learned experiences is referred to as *continual* or *lifelong learning*. In contrast to state-of-the-art deep learning models that typically rely on the full training set being available at once (see LeCun et al., 2015 for a review), lifelong learning systems must account for situations in which the training data become incrementally available over time. Effective models of lifelong learning are crucial in real-world conditions where an autonomous agent cannot be provided with all the necessary prior knowledge to interact with the environment and the direct access to previous experience is restricted (Thrun and Mitchell, 1995). Importantly, there may be no distinction between training and test phases, which requires the system to concurrently learn and timely trigger behavioral responses (Cangelosi and Schlesinger, 2015; Tani, 2016).

Lifelong machine learning represents a long-standing challenge due to *catastrophic forgetting* or *interference*, i.e., training a model with a new task leads to an abrupt decrease in the performance on previously learned tasks (McCloskey and Cohen, 1989). To overcome catastrophic forgetting, computational models must adapt their existing representations on the basis of novel sensory experience while preventing disruptive interference with previously learned representations. The extent to which a system must be flexible for learning novel knowledge and stable for preventing the disruption of consolidated knowledge is known as the *stability-plasticity dilemma*, which has been extensively studied for both computational and biological systems (e.g., Grossberg, 1980, 2007; Mermillod et al., 2013; Ditzler et al., 2015).

Neurophysiological evidence suggests distributed mechanisms of structural plasticity that promote lifelong memory formation, consolidation, and retrieval in multiple brain areas (Power and Schlaggar, 2016; Zenke et al., 2017a). Such mechanisms support the development of the human cognitive system on the basis of sensorimotor experiences over sustained time spans (Lewkowicz, 2014). Crucially, the brain must constantly perform two complementary tasks: (i) recollecting separate episodic events (specifics), and (ii) learning the statistical structure from the episodic events (generalities). The complementary learning systems (CLS) theory (McClelland et al., 1995; Kumaran et al., 2016) holds that these two interdependent operations are mediated by the interplay of the mammalian hippocampus and neocortex, providing the means for *episodic memory* (specific experience) and *semantic memory* (general structured knowledge). Accordingly, the hippocampal system exhibits quick learning of sparse representations from episodic experience which will, in turn, be transferred and integrated into the neocortical system characterized by a slower learning rate with more compact representations of statistical regularities.

Re-training a (deep) neural architecture from scratch in response to novel sensory input can require extensive computational effort. Furthermore, storing all the previously encountered data in lifelong learning scenarios has the general drawback of large memory requirements. Instead, Robins (1995) proposed *pseudo-rehearsal* (or *intrinsic replay*) in which previous memories are revisited without the need of explicitly storing data samples. *Pseudo-samples* are drawn from a probabilistic or generative model and replayed to the system for memory consolidation. From a biological perspective, the direct access to past experiences is limited or restricted. Therefore, the replay of hippocampal representations in the absence of external sensory input plays a crucial role in memory encoding (Carr et al., 2011; Kumaran et al., 2016). Memory replay is argued to occur through the reactivation of neural patterns during both sleep and awake states (e.g., free recall; Gelbard-Sagiv et al., 2008). Hippocampal replay provides the means for the gradual integration of knowledge into neocortical structures through the reactivation of recently acquired knowledge interleaved with the exposure to ongoing episodic experience (McClelland et al., 1995). Consequently, the periodic replay of previously encountered samples can alleviate catastrophic forgetting during incremental learning tasks, especially when the number of training samples for the different classes is unbalanced or when a sample is encountered only once (Robins, 1995).

A number of computational approaches have drawn inspiration from the learning principles observed in biological systems. Machine learning models addressing lifelong learning can be divided into approaches that regulate intrinsic levels of plasticity to protect consolidated knowledge, that dynamically allocate neural resources in response to novel experience, or that use complementary dual-memory systems with memory replay (see section 2). However, most of these methods are designed to address supervised learning on image datasets of very limited complexity such as MNIST (LeCun et al., 1998) and CIFAR-10 (Krizhevsky, 2009) while not scaling up to incremental learning tasks with larger-scale datasets of natural images and videos (Kemker et al., 2018; Parisi et al., 2018a). Crucially, such models do not take into account the temporal structure of the input which plays an important role in more realistic learning conditions, e.g., an autonomous agent learning from the interaction with the environment. Therefore, in contrast to approaches in which static images are learned and recognized in isolation, we focus on lifelong learning tasks where sequential data with meaningful temporal relations become progressively available over time.

In this paper, we propose a growing dual-memory (GDM) architecture for the lifelong learning of spatiotemporal representations from videos, performing continuous object recognition at an instance level (episodic knowledge) and at a category level (semantic knowledge). The architecture comprises two recurrent self-organizing memories that dynamically adapt the number of neurons and synapses: the episodic memory learns representations of sensory experience in an unsupervised fashion through input-driven plasticity, whereas the semantic memory develops more compact representations of statistical regularities embedded in episodic experience. For this purpose, the semantic memory receives neural activation trajectories from the episodic memory and uses task-relevant signals (annotated labels) to modulate levels of neurogenesis and neural update. Internally generated neural activity patterns in the episodic memory are periodically replayed to both memories in the absence of sensory input, thereby mitigating catastrophic forgetting during incremental learning. We conduct a series of experiments with the recently published Continuous Object Recognition (CORe50) benchmark dataset (Lomonaco and Maltoni, 2017). The dataset comprises 50 objects within 10 categories with image sequences captured under different conditions and containing multiple views of the same objects (indoors and outdoors, varying background, object pose, and degree of occlusion). We show that our model scales up to learning novel object instances and categories and that it outperforms current lifelong learning approaches in three different incremental learning scenarios.

## 2. Related work

The CLS theory (McClelland et al., 1995) provides the basis for computational frameworks that aim to generalize across experiences while retaining specific memories in a lifelong fashion. Early computational attempts include French (1997) who developed a dual-memory framework using pseudo-rehearsal (Robins, 1995) to transfer memories, i.e., the training samples are not explicitly kept in memory but drawn from a probabilistic model. However, there is no empirical evidence showing that this or similar contemporaneous approaches (see O'Reilly and Norman, 2002 for a review) scale up to large-scale image and video benchmark datasets. More recently, Gepperth and Karaoguz (2015) proposed two approaches for incremental learning using a modified self-organizing map (SOM) and a SOM extended with a short-term memory (STM). We refer to these two approaches as GeppNet and GeppNet+STM, respectively. In GeppNet, task-relevant feedback from a regression layer is used to select whether learning in the self-organizing hidden layer takes place. In GeppNet+STM, the STM is used to store novel knowledge which is occasionally played back to the GeppNet layer during sleep phases interleaved with training phases. This latter approach yields better performance and faster convergence in incremental learning tasks with the MNIST dataset. However, the STM has a limited capacity, thus learning new knowledge can overwrite old knowledge. In both cases, the learning process is divided into the initialization and the actual incremental learning phase. Furthermore, GeppNet and GeppNet+STM require storing the entire training dataset during training. Kemker and Kanan (2018) proposed the FearNet model for incremental class learning inspired by studies of memory recall and consolidation in the mammalian brain during fear conditioning (Kitamura et al., 2017). FearNet uses a hippocampal network capable of immediately recalling new examples, a PFC network for long-term memories, and a third neural network inspired by the basolateral amygdala for determining whether the system should use the PFC or hippocampal network for a particular example. FearNet consolidates information from its hippocampal network to its PFC network during sleep phases. Kamra et al. (2018) presented a similar dual-memory framework for lifelong learning that uses a variational autoencoder as a generative model for pseudo-rehearsal. Their framework generates a short-term memory module for each new task. However, prior to consolidation, predictions are made using an oracle, i.e., they know which module contains the associated memory.

Different methods have been proposed that are based on regularization techniques to impose constraints on the update of the neural weights. This is inspired by neuroscience findings suggesting that consolidated knowledge can be protected from interference via changing levels of synaptic plasticity (Benna and Fusi, 2016) and is typically modeled in terms of adding regularization terms that penalize changes in the mapping function of a neural network. For instance, Li and Hoiem (2016) proposed a convolutional neural network (CNN) architecture in which the network that predicts the previously learned tasks is enforced to be similar to the network that also predicts the current task by using knowledge distillation, i.e., the transferring of knowledge from a large, highly regularized model to a smaller model. This approach, known as learning without forgetting (LwF), has the drawbacks of highly depending on the relevance of the tasks and that the training time for one task linearly increases with the number of old tasks. Kirkpatrick et al. (2017) proposed elastic weight consolidation (EWC) which adds a penalty term to the loss function and constrains the weight parameters that are relevant to retain previously learned tasks. However, this approach requires a diagonal weighting over the parameters of the learned tasks which is proportional to the diagonal of the Fisher information metric, with synaptic importance being computed offline and limiting its computational application to low-dimensional output spaces. Zenke et al. (2017b) proposed to alleviate catastrophic forgetting by allowing individual synapses to estimate their importance for solving a learned task. Similar to Kirkpatrick et al. (2017), this approach penalizes changes to the most relevant synapses so that new tasks can be learned with minimal interference. In this case, the synaptic importance is computed in an online fashion over the learning trajectory in the parameter space.

In general, regularization approaches comprise additional loss terms for protecting consolidated knowledge which, with a limited amount of neural resources, leads to a trade-off on the performance of old and novel tasks. Other approaches expand the neural architecture to accommodate novel knowledge. Rusu et al. (2016) proposed to block any changes to the network trained on previous knowledge and expand the architecture by allocating novel sub-networks with a fixed capacity to be trained with the new information. This prevents catastrophic forgetting but leads the complexity of the architecture to grow with the number of learned tasks. Draelos et al. (2017) trained an autoencoder incrementally using the reconstruction error to show whether the older digits were retained. Their model added new neural units to the autoencoder to facilitate the addition of new MNIST digits. Rebuffi et al. (2017) proposed the iCaRL approach which stores example data points that are used along with new data to dynamically adapt the weights of a feature extractor. By combining new and old data, they prevent catastrophic forgetting but at the expense of a higher memory footprint.

The approaches described above are designed for the classification of static images, often exposing the learning algorithm to training samples in a random order. Conversely, in more natural settings, we make use of the spatiotemporal structure of the input. In previous research (Parisi et al., 2017), we showed that the lifelong learning of action sequences can be achieved in terms of prediction-driven neural dynamics with internal representations emerging in a hierarchy of recurrent self-organizing networks. The networks can dynamically allocate neural resources and update connectivity patterns according to competitive Hebbian learning by computing the input based on its similarity with existing knowledge and minimizing interference by creating new neurons whenever they are required. This approach has shown competitive results with batch learning methods on action benchmark datasets. However, the neural growth and update are driven by the minimization of the bottom-up reconstruction error and, thus, without taking into account top-down, task-relevant signals that can regulate the plasticity-stability balance. Furthermore, the model cannot learn in the absence of external sensory input, which leads to a non-negligible degree of disruptive interference during incremental learning tasks.

## 3. Proposed method

**Figure 1:**
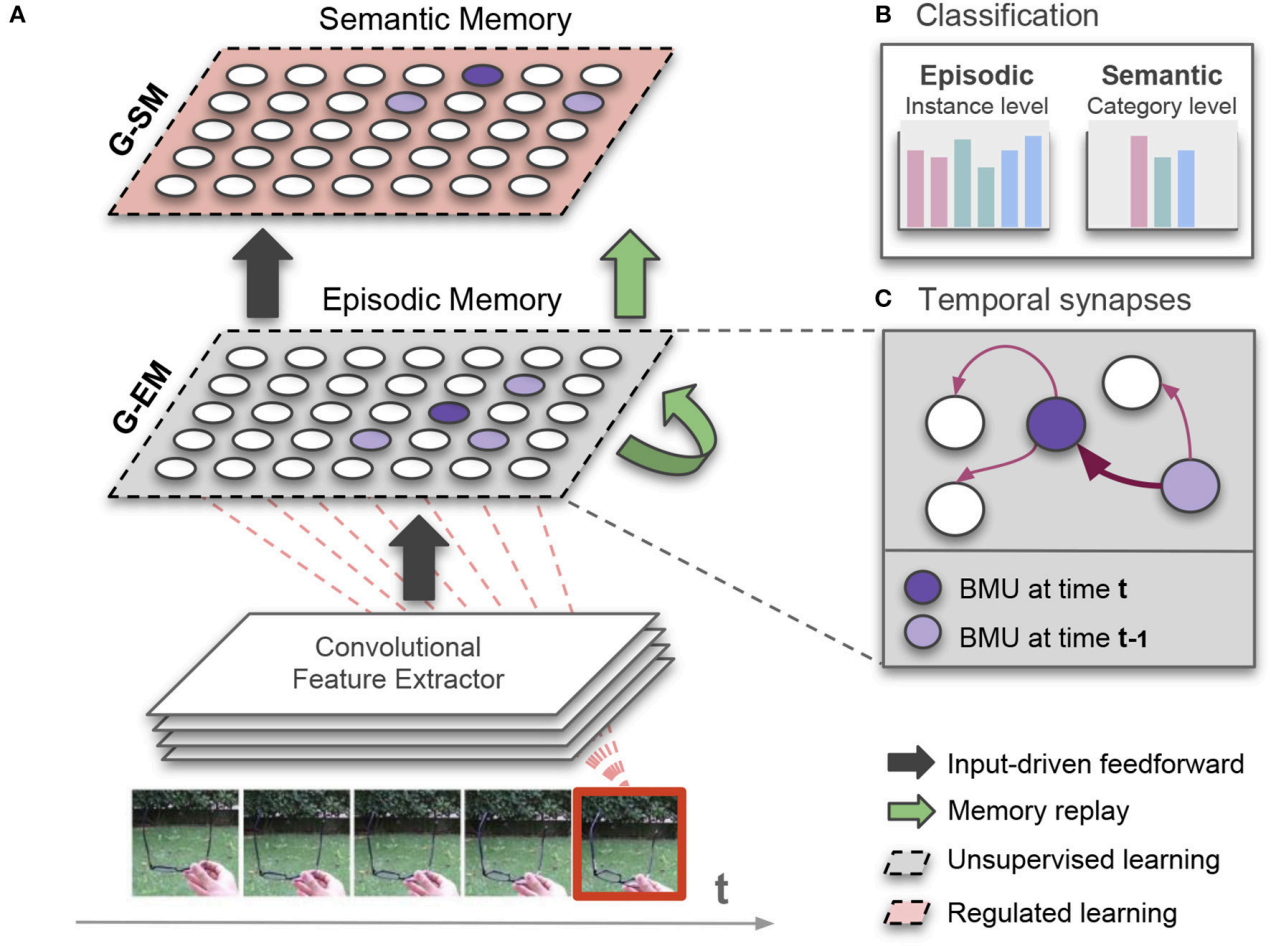
**(A)** Illustration of our growing dual-memory (GDM) architecture for lifelong learning. Extracted features from image sequences are fed into a growing episodic memory (G-EM) consisting of an extended version of the recurrent Grow-When-Required network (section 3.2). Neural activation trajectories from G-EM are feed-forwarded to the growing semantic memory (G-SM) that develops more compact representations of episodic experience (section 3.3). While the learning process of G-EM remains unsupervised, G-SM uses class labels as task-relevant signals to regulate levels of structural plasticity. After each learning episode, internally generated neural activation trajectories are replayed to both memories (green arrows; section 3.4); **(B)** The architecture classifies image sequences at instance level (episodic experience) and at category level (semantic knowledge). For the purpose of classification, neurons in G-EM and G-SM associatively learn histograms of class labels from the input (red dashed lines); **(C)** To enable memory replay in the absence of sensory input, G-EM is equipped with temporal synapses that are strengthened (thicker arrow) between consecutively activated best-matching units (BMU).

The proposed architecture with growing dual-memory learning (GDM) comprises a deep convolutional feature extractor and two hierarchically arranged recurrent self-organizing networks (Figure 1). Both recurrent networks are extended versions of the Gamma-GWR model (Parisi et al., 2017) that dynamically create new neurons and connections in response to novel sequential input. The growing episodic memory (G-EM) learns from sensory experience in an unsupervised fashion, i.e., levels of structural plasticity are regulated by the ability of the network to predict the spatiotemporal patterns given as input. Instead, the growing semantic memory (G-SM) receives neural activation trajectories from G-EM and uses task-relevant signals (input annotations) to modulate levels of neurogenesis and neural update, thereby developing more compact representations of statistical regularities embedded in episodic experience. Therefore, G-EM and G-SM mitigate catastrophic forgetting through self-organizing learning dynamics with structural plasticity, increasing information storage capacity in response to novel input.

The architecture classifies image sequences at an instance level (episodic experience) and a category level (semantic knowledge). Thus, each input sample carries two labels which are used for the classification task at the different levels of the network hierarchy. For the consolidation of knowledge over time in the absence of sensory input, internally generated neural activity patterns in G-EM are periodically replayed to both memories, thereby mitigating catastrophic forgetting during incremental learning tasks. For this purpose, G-EM is equipped with synapses that learn statistically significant neural activity in the temporal domain. As a result, sequence-selective neural activation trajectories can be generated and replayed after each learning episode without explicitly storing sequential input.

### 3.1. Gamma-GWR

The Gamma-GWR model (Parisi et al., 2017) is a recurrent extension of the Grow-When-Required (GWR) self-organizing network (Marsland et al., 2002) that embeds a Gamma memory (Principe et al., 1994) for representing short-term temporal relations. The Gamma-GWR can dynamically grow or shrink in response to the sensory input distribution. New neurons will be created to better represent the input and connections (synapses) between neurons will develop according to competitive Hebbian learning, i.e., neurons that co-activate will be connected to each other. The Gamma-GWR learns the spatiotemporal structure of the input through the integration of temporal context into the computation of the self-organizing network dynamics.

The network is composed of a dynamic set of neurons, *A*, with each neuron consisting of a weight vector **w**_*j*_ and a number *K* of context descriptors **c**_*j, k*_ (${\text{w}}_{j},{\text{c}}_{j,k}\in {\mathbb{R}}^{n}$). Given the input **x**(*t*) ∈ ℝ^*n*^, the index of the best-matching unit (BMU), *b*, is computed as:

(1) $$\begin{array}{r} b=\arg\underset{j\in A}{\min}\left(d_{j}\right), \end{array}$$

(2) $$\begin{array}{r} d_{j}=\alpha_{0}∥\text{x}\left(t\right)-{\text{w}}_{j}{∥}^{2}+\overset{K}{\underset{k=1}{\sum }}\;\alpha_{k}∥{\text{C}}_{k}\left(t\right)-{\text{c}}_{j,k}{∥}^{2}, \end{array}$$

(3) $$\begin{array}{r} {\text{C}}_{k}\left(t\right)=\beta \cdot {\text{w}}_{b}^{t-1}+\left(1-\beta \right)\cdot {\text{c}}_{b,k-1}^{t-1}, \end{array}$$

where ∥·∥^2^ denotes the Euclidean distance, α_*i*_ and β are constant factors that regulate the influence of the temporal context, ${\text{w}}_{b}^{t-1}$ is the weight vector of the BMU at *t*−1, and ${\text{C}}_{k}\in {\mathbb{R}}^{n}$ is the global context of the network with **C**_*k*_(*t*_0_) = 0.

The activity of the network, *a*(*t*), is defined in relation to the distance between the input and its BMU (Equation 2) as follows:

(4) $$\begin{array}{r} a\left(t\right)=\exp\left(-d_{b}\right), \end{array}$$

thus yielding the highest activation value of 1 when the network can perfectly predict the input sequence (i.e., *d*_*b*_ = 0). Furthermore, each neuron is equipped with a habituation counter *h*_*j*_ ∈ [0, 1] expressing how frequently it has fired based on a simplified model of how the efficacy of a habituating synapse reduces over time (Stanley, 1976). Newly created neurons start with *h*_*j*_ = 1. Then, the habituation counter of the BMU, *b*, and its neighboring neurons, *n*, iteratively decrease toward 0. The habituation rule (Marsland et al., 2002) for a neuron *i* is given by:

(5) $$\begin{array}{r} \Delta h_{i}=\tau_{i}\cdot \kappa \cdot \left(1-h_{i}\right)-\tau_{i}, \end{array}$$

with *i* ∈ {*b, n*} and where τ_*i*_ and κ are constants that control the monotonically decreasing behavior. Typically, *h*_*b*_ is decreased faster than *h*_*n*_ with τ_*b*_ > τ_*n*_.

The network is initialized with two neurons and, at each learning iteration, a new neuron is created whenever the activity of the network, *a*(*t*), in response to the input **x**(*t*) is smaller than a given insertion threshold *a*_*T*_. Furthermore, *h*_*b*_ must be smaller than a habituation threshold *h*_*T*_ in order for the insertion condition to hold, thereby fostering the training of existing neurons before new ones are added. The new neuron is created halfway between the BMU and the input. The training of the neurons is carried out by adapting the BMU *b* and the neurons *n* to which the *b* is connected:

(6) $$\begin{array}{r} \Delta {\text{w}}_{i}=\epsilon_{i}\cdot h_{i}\cdot \left(\text{x}\left(t\right)-{\text{w}}_{i}\right), \end{array}$$

(7) $$\begin{array}{r} \Delta {\text{c}}_{i,k}=\epsilon_{i}\cdot h_{i}\cdot \left({\text{C}}_{k}\left(t\right)-{\text{c}}_{i,k}\right), \end{array}$$

with *i* ∈ {*b, n*} and where ϵ_*i*_ is a constant learning rate (ϵ_*n*_ < ϵ_*b*_). Furthermore, the habituation counters of the BMU and the neighboring neurons are updated according to Equation (5). Connections between neurons are updated on the basis of neural co-activation, i.e., when two neurons fire together (BMU and second-BMU), a connection between them is created if it does not exist. Each connection has an age that increases at each learning iteration. The age of the connection between the BMU and the second-BMU is reset to 0, whereas the other ages are increased by a value of 1. The connections with an age greater than a given threshold can be removed, and neurons without connections can be deleted.

For the purpose of classification, an associative matrix *H*(*j, l*) stores the frequency-based distribution of sample labels during the learning phase, so that each neuron *j* stores the number of times that an input with label *l* had *j* as its BMU. As a result, the predicted label ξ_*j*_ for a neuron *j* can be computed as:

(8) $$\begin{array}{r} \xi_{j}=\arg\underset{l\in L}{\max}H\left(j,l\right), \end{array}$$

where *l* is an arbitrary label. Therefore, the unsupervised Gamma-GWR can be used for classification without requiring the number of label classes to be predefined.

### 3.2. Episodic memory

The learning process of growing episodic memory G-EM is unsupervised, thereby creating new neurons or updating existing ones to minimize the discrepancy between the sequential input and its neural representation. In this way, episodic memories can be acquired and fine-tuned iteratively through sensory experience. This is functionally consistent with hippocampal representations, e.g., in the dentate gyrus, which are responsible for pattern separation through the orthogonalization of incoming inputs supporting the auto-associative storage and retrieval of item-specific information from individual episodes (Yassa and Stark, 2011; Neunuebel and Knierim, 2014).

Given an input image frame, the extracted image feature vector (see section 4.1) is given as input to G-EM which recursively integrates the temporal context into the self-organizing neural dynamics. The spatial resolution of G-EM neurons can be tuned through the insertion threshold, *a*_*T*_, with a greater *a*_*T*_ leading to more fine-grained representations since new neurons will be created whenever *a*(*t*) < *a*_*T*_ (see Equation 4). The temporal depth is set by the number of context descriptors, *K*, with a greater *K* yielding neurons that activate for larger temporal windows (longer sequences), whereas the temporal resolution is set by the hyperparameters α and β (see Equations 2 and 3).

To enable memory replay in the absence of external sensory input, we extend the Gamma-GWR model by implementing temporal connections that learn trajectories of neural activity in the temporal domain. Such temporal connections are sequence-selective synaptic links which are incremented between two consecutively activated neurons (Parisi et al., 2016). Sequence selectivity driven by asymmetric connections has been argued to be a feature of the cortex (Mineiro and Zipser, 1998), where an active neuron pre-activates neurons encoding future patterns. Formally, when the two neurons *i* and *j* are consecutively activated at time *t*−1 and *t*, respectively, their temporal synaptic link *P*_(*i, j*)_ is increased by Δ*P*_(*i, j*)_ = 1. For each neuron *i*∈*A*, we can retrieve the next neuron *v* of a prototype trajectory by selecting

(9) $$\begin{array}{r} v=\arg\underset{j\in A\backslash i}{\max}P_{\left(i,j\right)}. \end{array}$$

Recursively generated neural activation trajectories can be used for memory replay (see section 3.4). During the learning phase, G-EM neurons will store instance-level label classes ξ^*I*^ for the classification of the input (see Equation 8). Furthermore, since trajectories of G-EM neurons are replayed to G-SM in the absence of sensory input, G-EM neurons will also store labels at a category label *l*^*C*^. Therefore, the associative matrix for each neuron *j* is of the form *H*(*j, l*^*I*^, *l*^*C*^).

### 3.3. Semantic memory

The growing semantic memory G-SM combines bottom-up drive from neural activity in G-EM and top-down signals (i.e., category-level labels from the input) to regulate structural plasticity levels. More specifically, the mechanisms of neurogenesis and neural weight update are regulated by the ability of G-SM to correctly classify its input. Therefore, while G-EM iteratively minimizes the discrepancy between the input sequences and their internal representations, G-SM will create new neurons only if the correct label of a training sample cannot be predicted by its BMU in G-SM. This is implemented as an additional constraint in the condition for neurogenesis so that new neurons are not created unless the predicted label of the BMU (Equation 8) does not match the input label.

G-SM receives as input activated neural weights from G-EM, i.e., the weight vector of a BMU in G-EM, ${\text{w}}_{b}^{\text{EM}}$, for a given input frame. As an additional mechanism to prevent novel sensory experience from interfering with consolidated representations, G-SM neurons are updated (Equations 6 and 7) only if the predicted label for the BMU in G-SM matches in the input label, i.e., if the BMU codes for the same object category as the input. In this way, the representations of an object category cannot be updated in the direction of the input belonging to a different category, which would cause disruptive interference.

As a result of hierarchical processing, G-SM neurons code for information acquired over larger temporal windows than neurons in G-EM. That is, one G-SM will fire for a number *K*^SM^ + 1 of neurons fired in G-EM (where *K*^SM^ is the temporal depth of G-SM neurons). Since G-EM neurons will fire for a number *K*^EM^ + 1 of input frames, G-SM neurons will code for a total of *K*^SM^ + *K*^EM^ + 1 input frames. This is consistent with established models of memory consolidation where neocortical representations code for information acquired over more extended time periods than the hippocampus (e.g., Kumaran and McClelland, 2012; Kumaran et al., 2016), thereby yielding a higher degree of temporal slowness.

Temporal slowness results from the statistical learning of spatiotemporal regularities, with neurons coding for prototype sequences of sensory experience. By using category-level signals to regulate neural growth and update, G-SM will develop more compact representations from episodic experience with neurons activating in correspondence of semantically-related input, e.g., the same neuron may activate for different instances of the same category and, because of the processing of temporal context, the same object seen from different angles. However, specialized mechanisms of slow feature analysis can be implemented that would yield invariance to complex input transformations such as view invariance (e.g., Berkes and Wiskott, 2005; Einhäuser et al., 2005). View invariance of objects is a prominent property of higher-level visual areas of the mammalian brain, with neurons coding for abstract representations of familiar objects rather than for individual views and visual features (Booth and Rolls, 1998; Karimi-Rouzbahani et al., 2017). Neurophysiological studies evidence that distributed representations in high-level visual regions of the neocortex (semantic) are less sparse than those of the hippocampus (episodic) and where related categories are represented by overlapping neural codes (Clarke and Tyler, 2014; Yamins et al., 2018).

### 3.4. Memory replay

Hippocampal replay provides the means for the gradual integration of knowledge into neocortical structures and is thought to occur through the reactivation of recently acquired knowledge interleaved with the exposure to ongoing experiences (McClelland et al., 1995). Although the periodic replay of previous data samples can alleviate catastrophic forgetting, storing all previously encountered data samples has the general drawback of large memory requirements and large retraining computational times.

In pseudo-rehearsal (or intrinsic replay), memories are drawn from a probabilistic or generative model and replayed to the system for memory consolidation (Robins, 1995). In our case, however, we cannot simply draw or generate isolated and randomly selected pseudo-samples from a given distribution since we must account for preserving the temporal structure of the input. Therefore, we generate pseudo-patterns in terms of temporally-ordered trajectories of neural activity. For this purpose, we propose to use the asymmetric temporal links of G-EM (section 3.2) to recursively reactivate sequence-selective neural activity trajectories (RNATs) embedded in the network. RNATs can be computed for each neuron in G-EM for a given temporal window and replayed to G-EM and G-SM after each learning episode triggered by external input stimulation.

For each neuron *j* in G-EM, we generate a RNAT, *S*_*j*_, of length λ = *K*^EM^ + *K*^SM^ + 1 as follows:

(10) $$\begin{array}{r} S_{j}=\langle {\text{w}}_{\text{s}\left(0\right)}^{\text{EM}},{\text{w}}_{\text{s}\left(1\right)}^{\text{EM}},\ldots ,{\text{w}}_{\text{s}\left(\lambda \right)}^{\text{EM}}\rangle , \end{array}$$

(11) $$\begin{array}{r} \text{s}\left(i\right)=\arg\underset{n\in A\backslash j}{\max}P_{\left(n,\text{s}\left(i-1\right)\right)},i\in \left[1,\lambda \right], \end{array}$$

where *P*_(*i, j*)_ is the matrix of temporal synapses (as defined by Equation 9) and s(0) = *j*. The class labels of the pseudo-patters in *S*_*j*_ can be retrieved according to Equation (8).

The set of generated RNATs from all G-EM neurons is replayed to G-EM and G-SM after each learning episode, i.e., a learning epoch over a batch of sensory observations. As a result of computing RNATs, sequence-selective prototype sequences can be generated and periodically replayed without the need of explicitly storing the temporal relations and labels of previously seen training samples. This is conceptually consistent with neurophysiological studies evidencing that hippocampal replay consists of the reactivation of previously stored patterns of neural activity occurring predominantly after an experience (Kudrimoti et al., 1999; Karlsson and Frank, 2009).

## 4. Experimental results

We perform a series of experiments evaluating the performance of the proposed GDM model in batch learning (section 4.2), incremental learning (section 4.3), and incremental learning with memory replay (section 4.4). We analyze and evaluate our model with the CORe50 dataset (Lomonaco and Maltoni, 2017; see section 4.1), a recently published benchmark for continuous object recognition from video sequences. We reproduce three experimental conditions defined by the CORe50 benchmark (section 4.5) showing that our model significantly outperforms state-of-the-art lifelong learning approaches. For the replication of these experiments, the source code of the GDM model is available as a repository^1^.

### 4.1. Feature extraction

**Figure 2:**
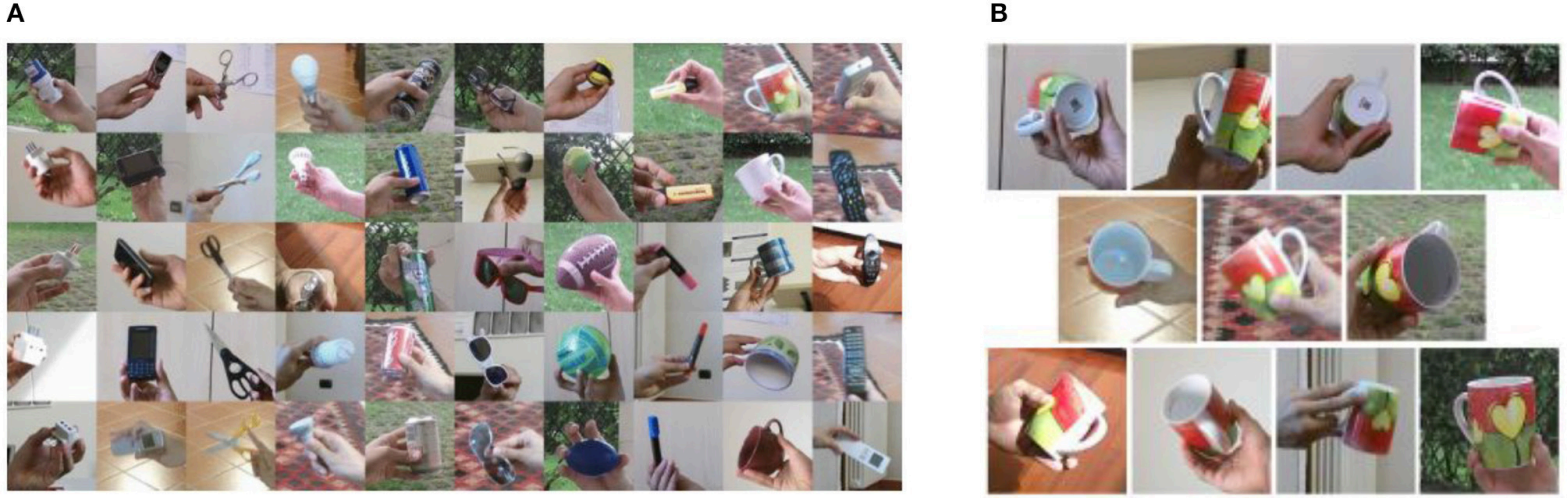
The CORe50 dataset designed for continuous object recognition: **(A)** Example frames of the 10 categories (columns) comprising 5 object instances each, **(B)** Example frames for one object instance from the 11 acquisition sessions showing different background, illumination, pose, and degree of occlusion. Adapted from Lomonaco and Maltoni (2017).

The CORe50 comprises 50 objects within 10 categories with image sequences captured under different conditions and multiple views of the same objects (varying background, object pose, and degree of occlusion; see Figure 2). Each object comprises a video sequence of approximately 15 s where the object is shown to the vision sensor held by a human operator. The video sequences were collected in 11 sessions (8 indoors, 3 outdoors) with a Kinect 2.0 sensor delivering RGB (1027 × 575) and depth images (512 × 242) at 20 frames per second (fps) for a total of 164,866 frames. For our experiments, we used 128 × 128 RGB images provided by the dataset at a reduced frame rate of 5hz. The movements performed by the human operator with the objects (e.g., rotation) are quite smooth and reducing the number of frames per second has not shown significant loss of information.

For a more direct comparison with the baseline results provided by Lomonaco and Maltoni (2017) who adopted the VGG model (Simonyan and Zisserman, 2014) pre-trained on the ILSVRC-2012 dataset (Russakovsky et al., 2014), our feature extraction module consists of the same pre-trained VGG model to which we applied a convolutional operation with 256 1 × 1 kernels on the output of the fully-connected hidden layer to reduce its dimensionality from 2048 to 256. Therefore, G-EM receives a 256-dimensional feature vector per sequence frame. Such compression of the feature vectors is desirable since the Gamma-GWR uses the Euclidean distance as a metric to compute the BMUs, which becomes weakly discriminant when the data are very high-dimensional or sparse (Parisi et al., 2015). Furthermore, it is expected that different pre-trained models may exhibit a slightly better performance than VGG, e.g., ResNet-50 (He et al., 2015; see Lomonaco and Maltoni, 2018 for ResNet-50 performance on CORe50). However, here we focus on showing the contribution of context-aware growing networks rather than comparing deep feature extractors.

### 4.2. Batch learning

In the batch learning strategy, we trained the architecture on the entire training data at once and subsequently tested its classification performance at instance and category level. Following the same evaluation scheme described by Lomonaco and Maltoni (2017), we used the samples from sessions #3, #7, #10 for testing and the samples from the remaining 8 sessions for training. We compare our results to the baseline provided by Lomonaco and Maltoni (2017) using fine-tuning on a pre-trained VGG network (VGG+FT). To better assess the contribution of the temporal context (TC) for the task of continuous object recognition, we performed batch learning experiments with 3 different model configurations:

- **GDM**: We trained the model using TC and tested it on novel sequences. For each input frame, an object instance and an object category are predicted.
- **GDM (No TC)**: We trained and tested the model without TC by setting *K* = 0, i.e., the computation of the BMU is reduced to $b=\arg\underset{j\in A}{\min}∥\text{x}\left(t\right)-{\text{w}}_{j}{∥}^{2}$.
- **GDM (No TC during test)**: We trained the model with TC but tested on single image frames by setting *K* = 0.

The training hyperparameters are listed in Table 1. Except for the insertion thresholds $a_{T}^{\text{EM}}$ and $a_{T}^{\text{SM}}$, the remaining parameters were set similar to Parisi et al. (2017) for the incremental learning of sequences. Larger insertion thresholds will lead to a larger number of neurons. However, the best classification performance will not be necessarily obtained by the largest number of neurons. In G-EM, the neural representation should be characterized by a sufficiently high spatiotemporal resolution for discriminating between similar object instances and replaying episodic experience in the absence of sensory input. Conversely, regulated unsupervised learning in G-SM will lead to a more compact, overlapping neural representation with a smaller number of neurons while preserving the ability to correctly classify its input. The number of context descriptors (*K*^EM^, *K*^SM^) is set to 2. This means that G-EM neurons will activate in correspondence of 3 image frames and G-SM neurons in correspondence of 3 G-EM neurons, i.e., a processing window of 5 frames (1s of video at 5fps). Additional experiments showed that increasing the number of context descriptors does not significantly improve the overall accuracy. This is because a small number of context descriptors will lead to learning short-term temporal relations which are useful for temporal slowness, i.e., neurons that activate for multiple similar views of the same object (where different views of the object are induced by object motion). Neurons with a higher temporal depth will learn longer-term temporal relations and, depending on the difference between the training and test set, training with longer sequences may result in the specialization of neurons to the sequences in the training set while failing to generalize. Therefore, convenient values for *K*^EM^ and *K*^SM^ can be selected according to different criteria and properties of the input, e.g., number of frames per second, smoothness of object motion, desired degree of neural specialization.

**Table 1:** Training hyperparameters for the G-EM and G-SM networks (batch and incremental learning).

**Hyperparameters**	**Value**
Insertion thresholds	$a_{T}^{\text{EM}}=0.3$, $a_{T}^{\text{SM}}=0.001$
Habituation counters	*h*_*T*_ = 0.1, τ_*b*_ = 0.3, τ_*n*_ = 0.1, κ = 1.05
Temporal depth	*K*^EM^ = 2, *K*^SM^ = 2
Temporal context	α = [0.67, 0.24, 0.09], β = 0.7
Learning rates	ϵ_*b*_ = 0.5, ϵ_*n*_ = 0.005

The classification performance for the 3 different configurations is summarized in Table 2, showing instance-level and category-level accuracy after 35 training epochs averaged across 5 learning trials in which we randomly shuffled the batches from different sessions. The best results were obtained by GDM using temporal context with an average accuracy of 79.43% (instance level) and 93.92% (category level), showing an improvement of 10.35 and 13.69%, respectively, with respect to the baseline results (Lomonaco and Maltoni, 2017). Without the use of temporal context, the accuracy is comparable to the baseline showing a marginal improvement of 1.34% (instance level) and 3.31% (category level). Our results demonstrate that learning the temporal relations of the input plays an important role for this dataset. Interestingly, dropping the temporal component during the test phase, i.e., using single image frames for testing on context-aware networks, shows a slightly better performance (2.14 and 3.78%, respectively) than training without temporal context. This is because trained neural weights embed some temporal structure of the training sequences and, consequently, the context-free computation of a BMU from a single input frame will still be matched to context-aware neurons.

**Table 2:** Comparison of batch learning performance for instance-level and category-level classification.

	**Accuracy (%)**	**Accuracy (%)**
**Approach**	**(Instances)**	**(Categories)**
VGG + FT (Lomonaco and Maltoni, 2017)	69.08	80.23
Proposed GDM (No TC)	70.42	83.54
Proposed GDM (No TC during test)	72.56	87.32
Proposed GDM	**79.43**	**93.92**

*We show the accuracy for the pre-trained VGG with fine-tuning (VGG+FT) and the proposed GDM for three different configurations: (i) growing networks with temporal context (TC), (ii) without TC, and (iii) without TC during test. Best results in bold*.

**Figure 3:**
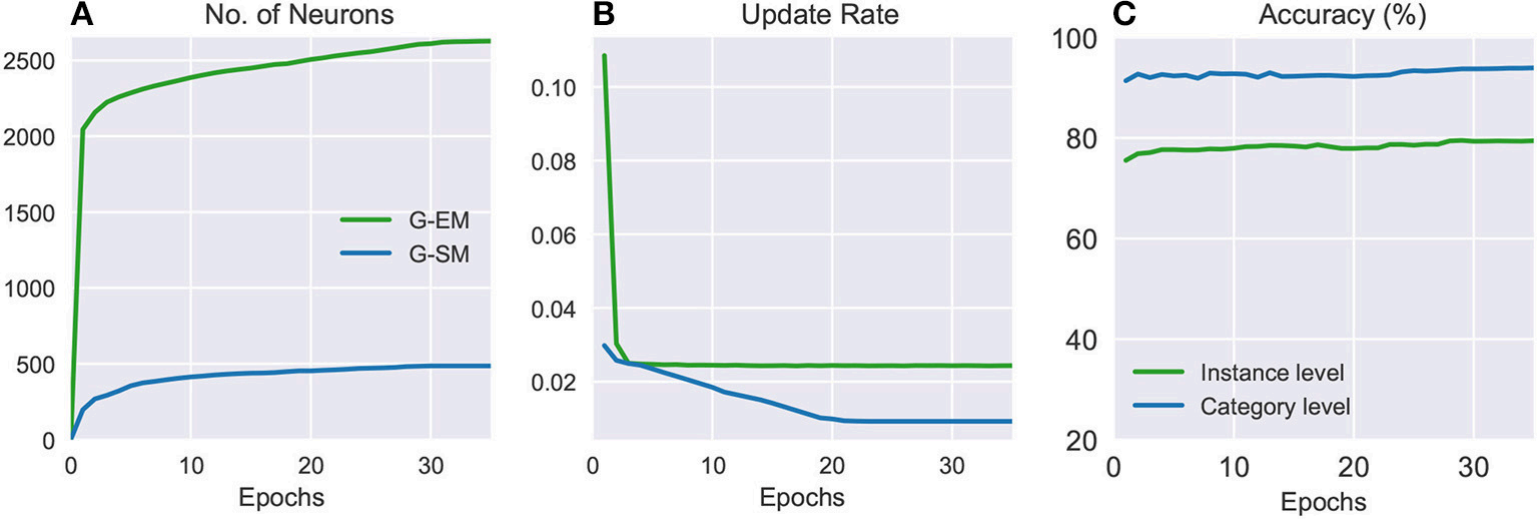
Batch learning on the CORe50: numbers of neurons **(A)**, update rates **(B)**, and classification accuracies **(C)** of G-EM and G-SM through 35 training epochs averaged across 5 learning trials.

Figure 3 shows the number of neurons, update rate, and classification accuracy for G-EM and G-SM (with temporal context) through 35 training epochs averaged across 5 learning trials. It can be seen that the average number of neurons created in G-EM is significantly higher than in G-SM (Figure 3A). This is expected since G-EM will grow to minimize the discrepancy between the input and its internal representation, whereas neurogenesis and neural update rate in G-SM are regulated by the ability of the network to predict the correct class labels of the input. The update rate (Figure 3B) is given by multiplying the fixed learning rate by the habituation counter of the neurons (ϵ_*i*_·*h*_*i*_), which shows a monotonically decreasing behavior. This indicates that, after a number of epochs, the created neurons become habituated to the input. Such a habituation mechanism has the advantage of protecting consolidated knowledge from being disrupted or overwritten by the learning of novel sensory experience, i.e., well-trained neurons will respond slower to changes in the distribution and the network will create new neurons to compensate for the discrepancy between the input and its representation.

### 4.3. Incremental learning

In the incremental learning strategy, the training samples of different object categories become progressively available over time, i.e., each mini-batch contains all the instances of an object category from all the 8 training sessions. Each category batch is shown once to the model and samples from that category are not shown again during the learning of new categories. Therefore, the model must incrementally learn new object instances and categories without forgetting previously learned ones. For a direct comparison with our previous experiment, the hyperparameters for the incremental learning experiment are the same as for the batch learning strategy (Table 1).

**Figure 4:**
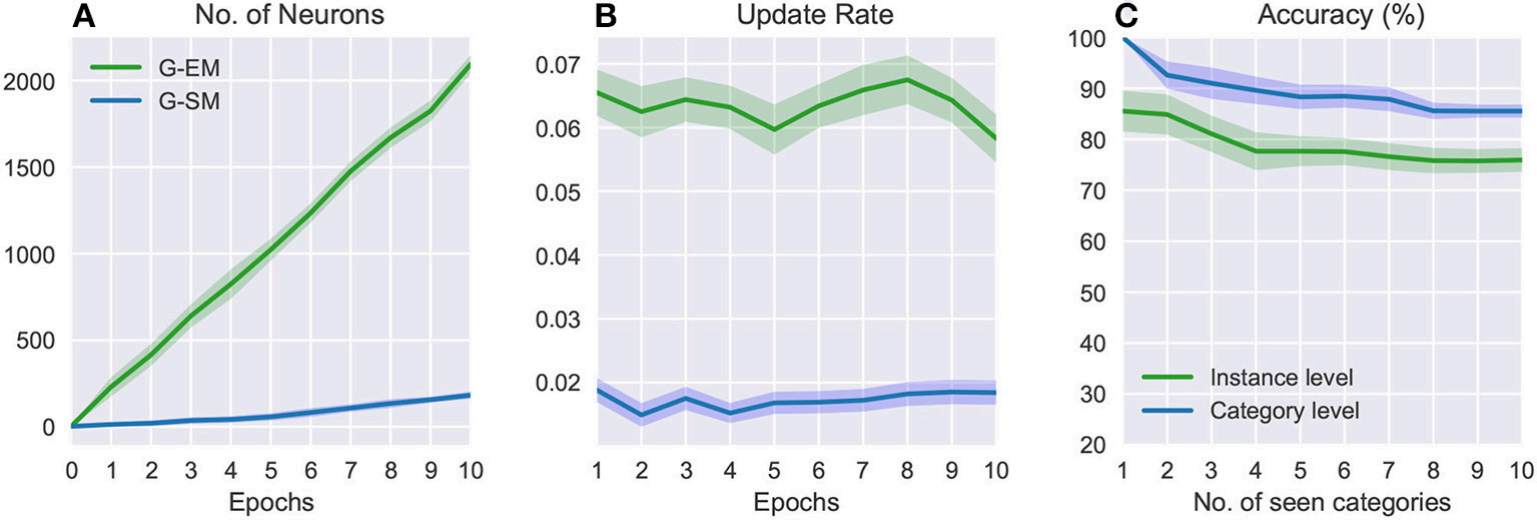
Incremental learning: numbers of neurons **(A)**, update rates **(B)**, and classification accuracies **(C)** over 10 categories averaged across 5 learning trials. The shaded areas show the standard deviation.

Figure 4 shows the number of neurons, update rate, and accuracy over 10 epochs (i.e., the 10 object categories) averaged across 5 runs of randomly shuffled object categories. The variance from the mean values (shaded areas in Figure 4) shows that the order of exposure to object categories can affect the final result. In general, the number of neurons increases over time (Figure 4A) and, in contrast to the batch learning strategy where neurogenesis is particularly strong during the initial training epochs (Figure 3A), in this case new neurons are progressively created in response to the exposure of the model to novel object classes. Similarly, the update rate for both networks (Figure 4B) does not monotonically decrease over time but rather stays quite stable in correspondence to novel sensory experience. Since newly created neurons are not well trained, the update rate will be higher at the moment of neural insertion and progressively decrease as the newly created neurons become habituated. The overall accuracy decreases with the number of object categories encountered, showing a higher sensitivity of the model with respect to the order in which the object categories are presented (Figure 4C). The average classification accuracy for the incremental learning strategy is 75.93%±2.23 (instance level) and 85.53%±1.35 (category level), showing a decrease of 3.5% and 8.39%, respectively, compared to the batch learning performance. This suggests that an additional mechanism such as memory replay is required to prevent the disruptive interference of existing representations.

**Figure 5:**
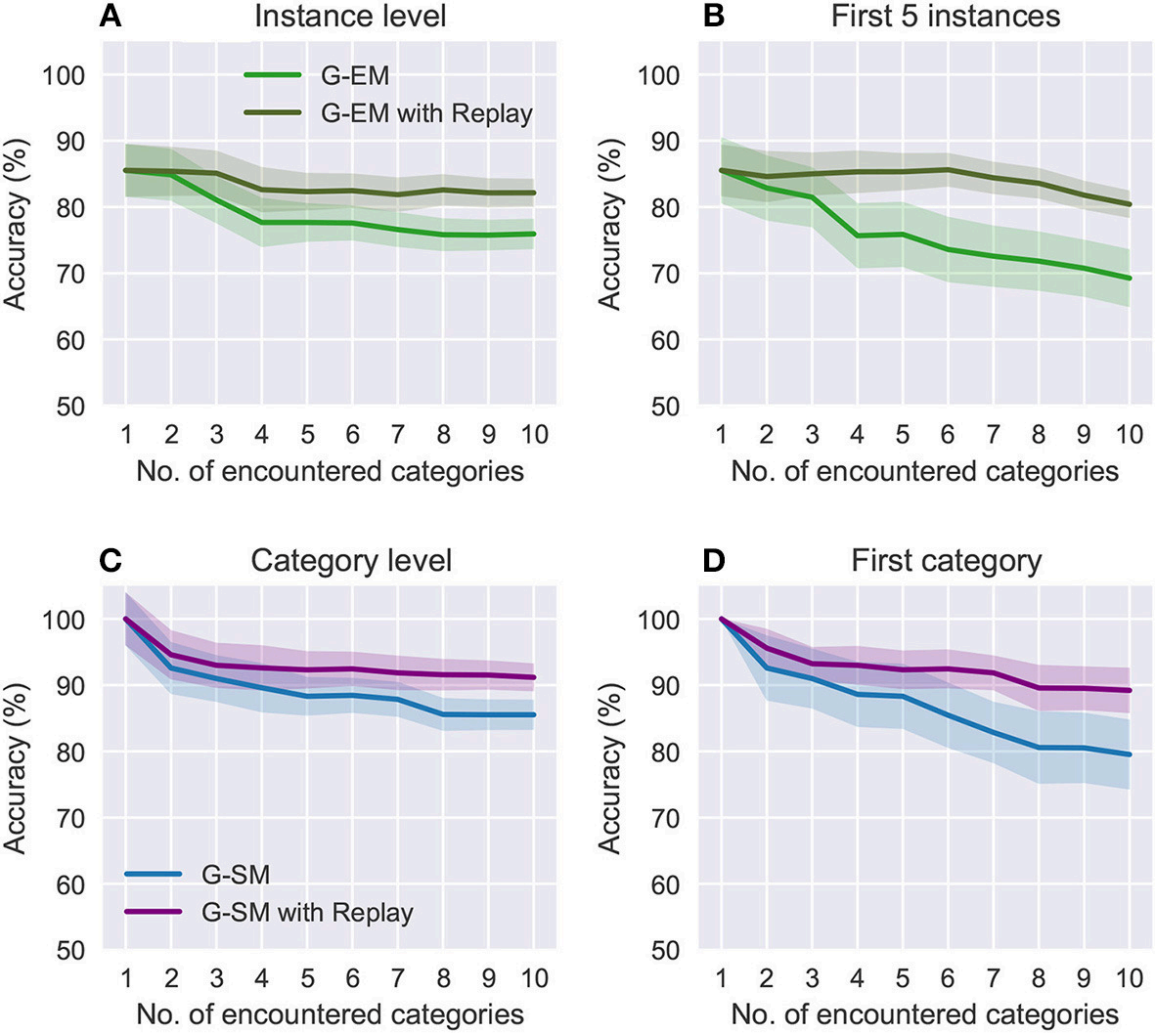
Comparison of the effects of forgetting during incremental learning with and without memory replay at an instance level **(A,B)** and category level **(C,D)**. Each category contains 5 instances. The plots show the average accuracies on the categories encountered so far **(A,C)** and the accuracies on the first encountered category **(B,D)** as further new categories are learned. The shaded areas show the standard deviation.

Figure 5 shows a comparison of the effects of forgetting during the incremental learning strategy in terms of the overall accuracy on the categories encountered so far and the accuracy on the first encountered category as new categories are learned. For the object instances, we compare the overall accuracy (Figure 5A) with the accuracy on the first 5 encountered instances (i.e., 1 category; see Figure 5B), showing that for the latter the accuracy drops to 69.25%±4.31 (compared to 75.93%±2.23). For the object categories (Figures 5C,D), the accuracy on the first encountered category drops to 79.53%±5.23 (compared to 85.53%±1.35). Overall, these results suggest that memory replay is an important feature for the reactivation of previously learned neural representations at the moment of learning from novel sensory experience with the goal to prevent that classes that have been encountered at early stages be forgotten over time.

### 4.4. Incremental learning with memory replay

In this learning strategy, we trained the model as described above with progressively available mini-batches containing 1 object category each. Here, however, after each learning episode (i.e., a training epoch over the mini-batch), the model generates a set of RNATs, *S*_*j*_ (Equations 10 and 11) from the G-EM neurons. Thus, the number of RNATs of length λ = 5 is equal to the number of neurons created by G-EM. The set of RNATs is replayed to G-EM and G-SM in correspondence of novel sensory experience to reinforce previously encountered categories. Since the growing self-organizing networks store the global temporal context, **C**_*k*_(*t*), over the training iterations (Equation 3) for learning the temporal structure of the input, each RNAT is fed into G-EM and G-SM as a single sample batch and the global temporal context is reset to zero after one epoch. It is expected that, by periodically replaying RNATs when new categories are encountered, knowledge representations will consolidate over time and, consequently, significantly alleviate catastrophic forgetting for sustained learning periods.

The benefit of using memory replay is shown in Figure 5 where we compare the overall accuracy on all the categories encountered so far to the accuracy on the first encountered category over the number of encountered categories. At an instance level (Figures 5A,B), incremental learning with memory replay improves the overall accuracy to 82.14%±2.05 (from 75.93%±2.23) and accuracy on the first 5 instances to 80.41%±1.35 (from 69.25%±2.01). At a category level (Figures 5C,D), the overall accuracy increases to 91.18%±0.25 (from 85.53%±1.35) and the accuracy on the first encountered category to 89.21%±3.37 (from 79.53%±5.23) Overall, our results support the hypothesis that replaying RNATs generated from G-EM mitigates the effects of catastrophic forgetting.

### 4.5. Continuous object recognition

We evaluate our model with the 3 incremental learning scenarios proposed by the CORe50 benchmark for the task of continuous object recognition:

**New Instances (NI)**: New instances of the same class and from different acquisition sessions become progressively available and are shown once to the model. Therefore, all the classes to be learned are known. For all the classes, the model is trained with the instances of a first session and subsequently with the remaining 7 sessions. (Here, the term *classes* is used for object categories.)

**New Classes (NC)**: Training samples from novel different classes become available over time, thus the model must deal with the learning of new classes without forgetting previously learned ones. Each training batch contains all the sequences of a small group of classes and memory replay is possible across batches. The first batch includes 10 objects while the remaining 8 batches contain 5 objects each. The test set includes samples from all the classes and the model is required to classify samples that have not been seen yet (except for the last evaluation step).

**New Instances and Classes (NIC)**: New instances and classes become available over time, requiring the model to consolidate knowledge about known classes and to learn new ones. The first batch includes 10 classes and the subsequent batches 5 classes each, with only one training sequence per class included in the batches. This scenario comprises 79 batches, maximizing the categorical representation in the first batch and randomly selecting the remaining 78 batches.

For each scenario, we compute the average accuracy over 10 configurations of randomly shuffled batches. The results for the NI, NC, and NIC scenarios compared to other approaches are listed in Table 3. It can be seen that our proposed method with memory replay produces state-of-the-art results for this benchmark dataset, showing an average accuracy of 87.94, 86.14, and 87.06% for the NI, NC, and NIC scenarios, respectively. These results represent a large increase in accuracy over 20% for each scenario with respect to the previous best results, i.e., a cumulative approach reported by Lomonaco and Maltoni (2017). The authors reported results using 3 methods with pre-trained CNN models and 128 × 128 images: (i) a naïve approach which consists of continuous stochastic gradient descent training as new batches become available, (ii) a proposed *CopyWeights with Re-init* (CWR) method that skips layers *fc6* and *fc7* of the CNN (for details, see Lomonaco and Maltoni, 2017; page 7), and (iii) a cumulative approach where the learning is carried out by considering the current batch and all the previous ones.

**Table 3:** Accuracy on the CORe50 incremental learning scenarios.

**Method**	**Avg. Acc. (%)**	**Std. Dev. (%)**
**NEW INSTANCES (NI)**		
Proposed GDM (with replay)	**87.94**	1.72
Proposed GDM	74.87	2.54
Cumulative (Lomonaco and Maltoni, 2017)	65.15	0.66
LwF (Li and Hoiem, 2016)	59.42*	2.71
EWC (Kirkpatrick et al., 2017)	57.40*	3.80
Naïve (Lomonaco and Maltoni, 2017)	54.69	6.18
**NEW CLASSES (NC)**		
Proposed GDM (with replay)	**86.14**	2.03
Proposed GDM	73.02	2.91
Cumulative	64.65	1.04
iCaRL (Rebuffi et al., 2017)	43.62*	0.66
CWR (Lomonaco and Maltoni, 2017)	42.32	1.09
LwF	27.60*	1.70
EWC	26.22*	1.18
Naïve	10.75	0.84
**NEW INSTANCES AND CLASSES (NIC)**		
Proposed GDM (with replay)	**87.06**	2.13
Proposed GDM	72.57	2.96
Cumulative	64.13	0.88
CWR	29.56	0
LwF	28.94*	4.30
EWC	28.31*	4.30
Naïve	19.39	2.90

Results denoted with * indicate the re-implementation of the method by Lomonaco and Maltoni (2017). Best results in bold.

Ours and previously reported experiments show that lifelong learning is a very challenging task and that the overall performance of some approaches can differ significantly according to the specific learning strategy. Furthermore, a more direct comparison of the model's behavior is hindered by the fact that the other methods do not comprise recurrent neural dynamics that account for learning the temporal structure of the input which, in this case, is a clear advantage (see Table 2) since the temporal relations of the input can be exploited for more robust learning and prediction.

The experiments reported for all the 3 incremental learning scenarios were conducted with the test set containing samples from all the seen classes (except for the last evaluation step). Such an evaluation scheme was selected to keep the test set consistent across all the scenarios (Lomonaco and Maltoni, 2017). However, in a more realistic lifelong learning scenario, the model should be able to deal with unknown classes during sequence retrieval. In our case, the model will always predict an output label in correspondence to a retrieved sequence. Instead, it would be convenient to design a novelty detection mechanism for unseen classes so that the system will output a predicted label provided that the input sequence produces a sufficient level of neural activity (Marsland et al., 2002; Parisi et al., 2015).

## 5. Discussion

### 5.1. Summary

We proposed a growing dual-memory architecture with self-organizing networks for the lifelong learning of spatiotemporal representations from image sequences. The GDM model can perform continuous object recognition at an instance level (episodic experience) and at a category level (semantic knowledge). We introduced the use of recurrent self-organizing networks, in particular of extended versions of the Gamma-GWR, to model the interplay of two complementary learning systems: an episodic memory, G-EM, with the task of learning fine-grained spatiotemporal representations from sensory experience and a semantic memory, G-SM, for learning more compact representations from episodic experience. With respect to previously proposed dual-memory learning systems, our contribution is threefold. First, in contrast to the predominant approach of processing static images independently, we implement recurrent self-organizing memories for learning the spatiotemporal structure of the input. Second, as a complementary mechanism to unsupervised growing networks, we use task-relevant signals to regulate structural plasticity levels in the semantic memory, leading to the development of more compact representations from episodic experience. Third, we model memory replay as the periodic reactivation of neural activity trajectories from temporal synaptic patterns embedded in an episodic memory. Our experiments show that the proposed GDM model significantly outperforms state-of-the-art lifelong learning methods in three different incremental learning tasks with the CORe50 benchmark dataset.

### 5.2. Growing recurrent networks with memory replay

The use of growing networks leads to the dynamic allocation of additional neurons and connections in response to novel sensory experience. In particular, the Gamma-GWR (Parisi et al., 2017) provides the basic mechanism for growing self-organizing memories with temporal context for learning the spatiotemporal structure of the input in an unsupervised fashion. Different models of neural network self-organization have been proposed that resemble the dynamics of Hebbian learning and plasticity (Fritzke, 1995; Kohonen, 1995; Marsland et al., 2002), with neural map organization resulting from unsupervised statistical learning. For instance, in the traditional self-organizing feature map and its dynamic variant (e.g., Kohonen, 1995; Rougier and Boniface, 2011, the number of neurons is pre-defined. Empirically selecting a convenient number of neurons can be tedious for networks with recurrent dynamics, especially when dealing with non-stationary input distributions (Strickert and Hammer, 2005). To alleviate this issue, growing self-organizing networks for temporal processing have been proposed, for instance the Gamma-GNG (Estévez and Vergara, 2012) that equips neurons with a temporal context. However, the Gamma-GNG grows at a constant, pre-defined interval and does not consider whether previously created neurons have been well trained before creating new ones. This will lead to scalability issues if the selected interval is too short or, conversely, to an insufficient number of neurons if the interval is too large. Therefore, we extended the Gamma-GWR which can quickly react to changes in the input distribution and can create new neurons whenever they are required.

From a biological perspective, there has been controversy over whether in human adults detectable amounts of new neurons can grow. Recent research has suggested that hippocampal neurogenesis drops sharply in children (Sorrells et al., 2018) and becomes undetectable in adulthood, whereas other studies suggest that hippocampal neurogenesis sustains human-specific cognitive function throughout life (Boldrini et al., 2018). Neurophysiological studies evidence that, in addition to neurogenesis, synaptic rewiring by structural plasticity has a significant contribution on memory formation in adults (Knoblauch et al., 2014; Knoblauch, 2017), with a major role of structural plasticity in increasing information storage efficiency in terms of space and energy demands. While the mechanisms for creating new neurons and connections in the Gamma-GWR do not resemble biologically plausible mechanisms of neurogenesis and synaptogenesis (e.g., Eriksson et al., 1998; Ming and Song, 2011; Knoblauch, 2017), the GWR learning algorithm represents an efficient computational model that incrementally adapts to non-stationary input. Crucially, the GWR model creates new neurons whenever they are required and only after the training of existing ones. The neural update rate decreases as the neurons become more habituated, which has the effect of preventing that noisy input interferes with consolidated neural representations. Alternative theories suggest that an additional function of hippocampal neurogenesis is the encoding of time for the formation of temporal associations in memory (Aimone et al., 2006, 2009), e.g., in terms of temporal clusters of long-term episodic memories. This represents an interesting research direction for the modeling of temporal associations in the Gamma-GWR.

For mitigating catastrophic forgetting during incremental learning tasks, the proposed model generates recurrent neural activity trajectories (RNATs; Equation 10) after each learning episode. The set of generated RNATs is periodically replayed to both networks in correspondence of novel sensory experience for the consolidation of knowledge over time. This is consistent with biological evidence suggesting that the reactivation of hippocampal representations and their frequent replay to the neocortex are crucial for memory consolidation and retrieval (see Carr et al., 2011 for a review). The process of replaying previously seen data without explicitly storing data samples is referred to as intrinsic replay (Robins, 1995) and has the advantage of fewer memory requirements with respect to explicitly storing training samples. In our approach, the episodic memory G-EM embeds the temporal structure of the input through the implementation of temporal synapses that are strengthened between consecutively activated neurons (Equation 9). Therefore, RNATs comprise prototype sequence snapshots that can be generated without the need of explicitly storing the training sequences. Our reported results show that the use of RNATs yields a significantly improved overall accuracy during incremental learning.

In this work, we have focused on regulating the mechanisms of neurogenesis and neural update, whereas we have not investigated the removal of old connections and isolated neurons. At each learning iteration of the Gamma-GWR, old connections exceeding a given age threshold and neurons without connections can be deleted. Removing a neuron from the network means that the knowledge coded by that unit is permanently forgotten. Therefore, a convenient maximum age of connections must be set to avoid catastrophic forgetting. In incremental learning scenarios, it is non-trivial to define a convenient age threshold for connections to be removed since data samples become available over time and neurons coding for consolidated knowledge might not fire for a large number of iterations. Mechanisms of intrinsic memory replay as modeled in this paper could be used to prevent the deletion of consolidated knowledge. For instance, the periodic replay of episodic representations would prevent the networks from deleting relevant knowledge also when external sensory input does not activate those representations for sustained periods of time.

Conceptual similarities can be found between our model and the adaptive resonance theory (ART) in which neurons are iteratively adapted to a non-stationary input distribution in an unsupervised fashion and new neurons can be added in correspondence of dissimilar input (see Grossberg, 2012 for a review). The primary intuition of the ART model is that learning occurs via the interaction of top-down and bottom-up processes, where top-down expectations act as memory templates or prototypes which are compared to bottom-up sensory observations. Similar to the GWR's activation threshold, the ART model uses a vigilance parameter to produce fine-grained or more general memories. Despite its inherent ability to mitigate catastrophic forgetting during incremental learning, it has been noted that the results of some variants of the ART model depend significantly upon the order in which the training data are processed. However, an extensive evaluation with recent lifelong learning benchmarks has not been reported. Therefore, ART-based models represent an additional complementary approach to growing self-organizing models.

## 6. Conclusion

Lifelong learning represents a fundamental but challenging component of artificial systems and autonomous agents. Despite significant advances in this direction, current models of lifelong learning are far from providing the flexibility, robustness, and scalability exhibited by biological systems. In this paper, we contribute to extending dual-memory models for the processing of sequential input which represents more realistic experimental settings compared to learning from static image datasets. In the future, it would be interesting to extend this model to the multisensory domain, e.g., where neural representations can be continually learned from audio-visual streams (Parisi et al., 2016, 2018b). The proposed architecture can be considered a further step toward more flexible lifelong learning methods that can be deployed in embodied agents for incrementally acquiring and refining knowledge over sustained periods through the active interaction with the environment.

## Author contributions

GP, JT, CW, and SW designed the experiments. GP wrote the paper. JT, CW, and SW revised the paper.

### Conflict of interest statement

The authors declare that the research was conducted in the absence of any commercial or financial relationships that could be construed as a potential conflict of interest.

## Footnotes

^1^GDM model: https://github.com/giparisi/GDM
